# GPNMB Extracellular Fragment Protects Melanocytes from Oxidative Stress by Inhibiting AKT Phosphorylation Independent of CD44

**DOI:** 10.3390/ijms221910843

**Published:** 2021-10-07

**Authors:** Qianqian Wang, Yasutaka Kuroda, Lingli Yang, Sylvia Lai, Yukiko Mizutani, Arunasiri Iddamalgoda, Jiao Guo, Asako Yamamoto, Daiki Murase, Yoshito Takahashi, Leihong Xiang, Shintaro Inoue, Daisuke Tsuruta, Ichiro Katayama

**Affiliations:** 1 Department of Pigmentation Research and Therapeutics, Graduate School of Medicine, Osaka City University, Osaka 5450051, Japan; 13621630785@163.com (Q.W.); kuroda.yasutaka@kao.com (Y.K.); s-lai@l.wdb-eu.com (S.L.); guojiao1986@gmail.com (J.G.); yamamoto.asako.1021@gmail.com (A.Y.); murase.daiki@kao.com (D.M.); takahashi.yoshito@kao.com (Y.T.); espaikoffice@gmail.com (I.K.); 2 Department of Dermatology, Huashan Hospital, Fudan University, Shanghai 200040, China; flora_xiang@vip.163.com; 3 Biological Science Research Laboratories, Kao Corporation, Kanagawa 2500002, Japan; 4 Department of Cosmetic Health Science, Gifu Pharmaceutical University, Gifu 5011196, Japan; mizutani@gifu-pu.ac.jp (Y.M.); arunasiri@ichimaru.co.jp (A.I.); inoshin@gifu-pu.ac.jp (S.I.); 5 Department of Research and Development, Ichimaru Pharcos Co. Ltd., Motosu, Gifu 5010475, Japan; 6 Department of Dermatology, Graduate School of Medicine, Osaka City University, Osaka 5458585, Japan; dtsuruta@med.osaka-cu.ac.jp

**Keywords:** GPNMB, oxidative stress, rhododendrol, vitiligo, AKT

## Abstract

Glycoprotein non-metastatic melanoma protein B (GPNMB) is a type I transmembrane glycoprotein that plays an important role in cancer metastasis and osteoblast differentiation. In the skin epidermis, GPNMB is mainly expressed in melanocytes and plays a critical role in melanosome formation. In our previous study, GPNMB was also found to be expressed in skin epidermal keratinocytes. In addition, decreased GPNMB expression was observed in the epidermis of lesional skin of patients with vitiligo. However, the exact role of keratinocyte-derived GPNMB and its effect on vitiligo is still unknown. In this study, we demonstrated that GPNMB expression was also decreased in rhododendrol-induced leukoderma, as seen in vitiligo. The extracellular soluble form of GPNMB (sGPNMB) was found to protect melanocytes from cytotoxicity and the impairment of melanogenesis induced by oxidative stress. Furthermore, the effect of rGPNMB was not altered by the knockdown of CD44, which is a well-known receptor of GPNMB, but accompanied by the suppressed phosphorylation of AKT but not ERK, p38, or JNK. In addition, we found that oxidative stress decreased both transcriptional GPNMB expression and sGPNMB protein expression in human keratinocytes. Our results suggest that GPNMB might provide novel insights into the mechanisms related to the pathogenesis of vitiligo and leukoderma.

## 1. Introduction

Vitiligo and chemical leukoderma are acquired hypopigmented skin disorders. Vitiligo causes patchy depigmentation due to the loss of functional epidermal melanocytes. The global prevalence of vitiligo currently ranges between 0.5% and 2% [1]. Although the exact underlying molecular mechanism of vitiligo is not fully understood, it is known to involve a complex interaction of multiple processes, such as the accumulation of genetic and epigenetic changes that may increase the sensitivity of melanocytes to injury or destruction by excessive UV irradiation, oxidative stress, chemical damage, and inflammatory factors [1]. Several tyrosine analogs, including 4-[4-hydroxyphenol]-2-butanol (rhododendrol; RD), induce chemical leukoderma or chemical-induced vitiligo [2]. RD-containing skin-whitening cosmetics induce chemical leukoderma, which occurs at the sites of use, and the repigmentation of part or all of the affected area is evident after discontinuation [3]. However, RD-induced leukoderma rarely presents with symptoms similar to those of vitiligo and induces concomitant vitiligo [3].

Glycoprotein non-metastatic melanoma protein B (GPNMB) is a type-I glycoprotein that was first identified in dendritic cells as a cell-associated transmembrane protein, dendritic cell-associated, heparin sulfate proteoglycan-dependent integrin ligand (DC-HIL), which promotes RGD-dependent cell adhesion [4]. It is also known as osteoactivin and hematopoietic growth factor-inducible neurokinin-1 and is widely expressed in various tissues, including the skin, brain, thymus, skeletal muscle, and bone [5,6,7,8,9]. Recently, GPNMB has been found to be widely expressed in various types of cells, including melanocytes, macrophages, dendritic cells, and various cancer cells, and is localized in the plasma membrane, melanosomes, and endosomal lysosomal compartment in the cytoplasm [10,11,12]. GPNMB contains three domains, including a long extracellular domain, a single transmembrane domain, and a relatively short cytoplasmic tail. The extracellular part of GPNMB is composed of an RGD motif that binds to integrin, maintaining cell–cell adhesion, and an Ig-like polycystic kidney disease domain involved in protein–protein and protein–carbohydrate interactions [11]. The extracellular fragment of GPNMB is cleaved by ADAM10 on the plasma membrane and secreted into the extracellular space [9,13].

GPNMB is important for the invasion and metastasis of several cancers [11] and plays diverse roles in normal cells, such as T-cell inactivation [14] and the promotion of the specialization of osteoclasts and osteoblasts [15,16]. Moreover, high GPNMB protein levels were observed in the cerebrospinal fluid, serum, and spinal cord of patients with amyotrophic lateral sclerosis [17]. Other studies have shown that the soluble form of GPNMB (sGPNMB) mediates signal transduction via cell surface proteins, such as CD44, as receptors for sGPNMB, showing anti-inflammatory or neuroprotective effects [18,19].

In the skin epidermis, GPNMB has been reported to be mainly expressed in melanocytes and regulated by microphthalmia-associated transcription factor (MITF), playing a critical role in pigmentation [20]. In melanocytes, GPNMB shares significant amino acid sequence homology with the melanosome protein Pmel-17 (25% amino acid sequence homology); It is present in all stages (I–IV) of melanosomes and is essential for the formation of melanosomes [10,21]. GPNMB functions as an adhesion protein between melanocytes and keratinocytes through integrin [22]. Until recently, the expression and function of GPNMB in epidermal keratinocytes have remained unclear. Tomihari et al. [22] showed that GPNMB was expressed in keratinocytes, while Hoashi et al. [10] showed that GPNMB was exclusively expressed in skin melanocytes, but not in keratinocytes or fibroblasts. Recently, we demonstrated that GPNMB was expressed in the basal keratinocytes of normal skin and nevus depigmentosus skin, but not in vitiligo skin [9]. However, as yet there is no detailed information on the involvement of keratinocyte-derived GPNMB in the pathophysiology of vitiligo and chemical leukoedema. This study was designed to assess the role of decreased epidermal GPNMB expression in vitiligo pathogenesis with a focus on melanocytes. Here, we report that melanocytes can be protected by extracellular GPNMB under oxidative stress conditions and that GPNMB expression was also decreased by oxidative stress in keratinocytes.

## 2. Results

### 2.1. Recombinant Extracellular Fragment of GPNMB (rGPNMB) Protects Melanocytes from Oxidative Stress and Rhododendrol Toxicity

**Figure 1:**
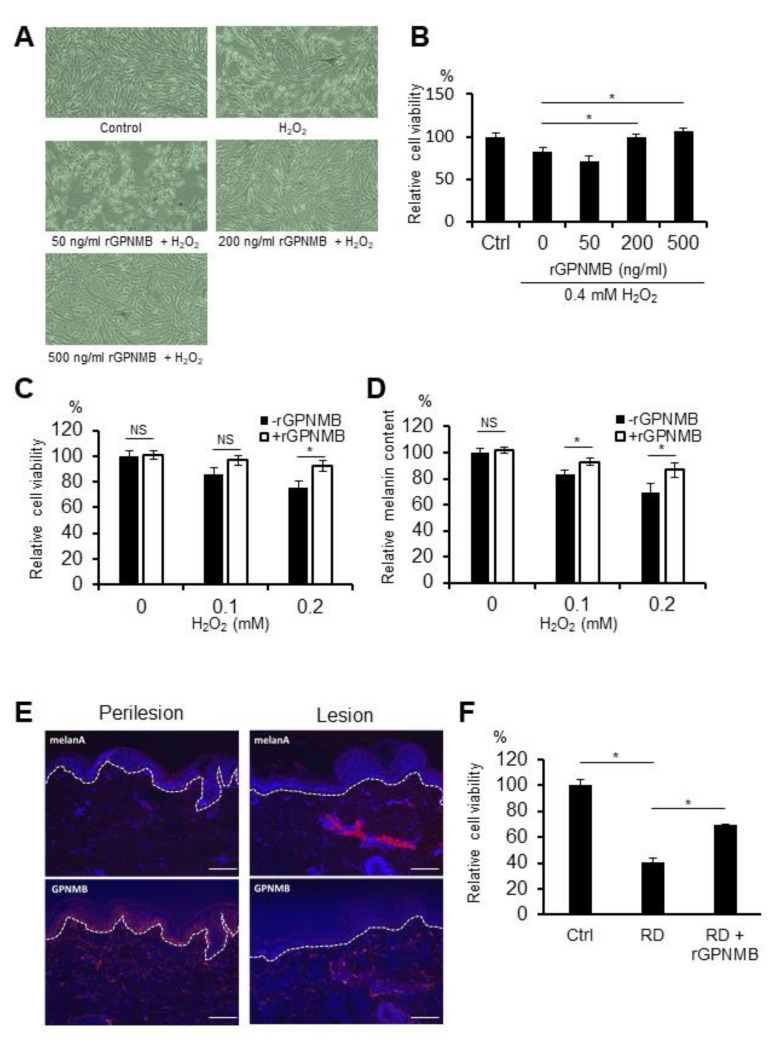
rGPNMB decreased the sensitivity to oxidative stress in melanocytes. (**A**,**B**) HEM-MP cells were treated with the indicated concentration of rGPNMB for 24 h and then treated with 0.4 mM of H_2_O_2_ for another 24 h. (**C**,**D**) HEM-MP cells were co-cultured with 500 ng/mL of rGPNMB for 24 h and then treated with 0.1 or 0.2 mM of H_2_O_2_ for another 8 d. (**E**) Skin samples collected from the perilesion and lesion of a patient with rhododendrol-induced leukoderma were immunostained using anti-human GPNMB antibody. GPNMB was stained red (lower panel), Melan-A was stained red (upper panel), and nucleus was stained blue. Scale bar = 100 μm. (**F**) HEM-MP cells were treated with 500 ng/mL of rGPNMB for 24 h and then treated with 1.5 mM of RD for another 24 h. (**A**) Cell shape was observed under bright-field microscopy. (**B**,**C**,**F**) Cell viability was quantified. (**D**) Melanin content in cell lysates was quantified. (**B**) * *p* < 0.05 (one-way ANOVA with Dunnett’s test). (**C,D**) * *p* < 0.05 (unpaired student’s *t*-test). NS: not significant. (**F**) * *p* < 0.05 (one-way ANOVA with Tukey’s test).

In vitiligo epidermis, GPNMB expression is diminished in basal keratinocytes [9]. To evaluate whether sGPNMB derived from keratinocytes can affect the function of melanocytes, we added the recombinant extracellular fragment of GPNMB (rGPNMB) to oxidative stress-bearing melanocytes. Hydrogen peroxide (H_2_O_2_) is currently the most widely used oxidative stress inducer. In our own research and the studies of others, a H_2_O_2_ concentration of 0.1 to 0.4 mM was found to be sub-toxic or toxic in melanocytes [23,24]. Human epidermal melanocytes from a moderately pigmented donor (HEM-MP) were stimulated with rGPNMB at the indicated concentration for 24 h and then treated with 0.4 mM of H_2_O_2_ for another 24 h. On the one hand, the morphology of cultured melanocytes changed under H_2_O_2_-exposed conditions (Figure 1A). On the other hand, rGPNMB protected melanocytes from oxidative stress at 200 and 500 ng/mL (Figure 1A,B). Furthermore, HEM-MP cells were co-cultured with 500 ng/mL of rGPNMB for 24 h and then treated with low H_2_O_2_ concentrations (0.1 mM and 0.2 mM) for another 8 d. The results showed that 500 ng/mL of rGPNMB was able to significantly protect melanocytes from oxidative stress, both in terms of cell viability (Figure 1C) and melanin production (Figure 1D). RD-induced leukoderma is a symptom similar to vitiligo [3], and RD metabolites (RD-quinone and RD-melanin) augment melanocyte toxicity via oxidative stress [25]. Therefore, we investigated GPNMB expression in the epidermis with RD-induced leukoderma and the protective function of GPNMB in RD-exposed melanocytes. GPNMB expression was significantly decreased in the lesional epidermis but not in the perilesional epidermis of patients with RD-induced leukoderma (Figure 1E). In addition, rGPNMB was found to significantly protect melanocytes from rhododendrol toxicity in a cell viability assay (Figure 1F).

### 2.2. srGPNMB Protects Melanocytes from Oxidative Stress via an NRF2-Independent Pathway

The NRF2/HO-1 pathway is involved in anti-oxidative responses. To explore the effect of sGPNMB on oxidative stress and the NRF2/HO-1 pathway in melanocytes, we analyzed the expression levels of antioxidant response-related proteins, including NRF2 and HO-1. These proteins were not found to be altered in rGPNMB- and H_2_O_2_-exposed HEM-MPs (Figure S1). The results showed that rGPNMB protected the melanocytes from oxidative stress, which may not be related to the enhancement of the antioxidant ability of melanocytes by the activation of the NRF2/HO-1 signaling pathway.

### 2.3. CD44 Knockdown Does Not Affect the Protective Effect of rGPNMB Against Oxidative Stress in Melanocytes

**Figure 2:**
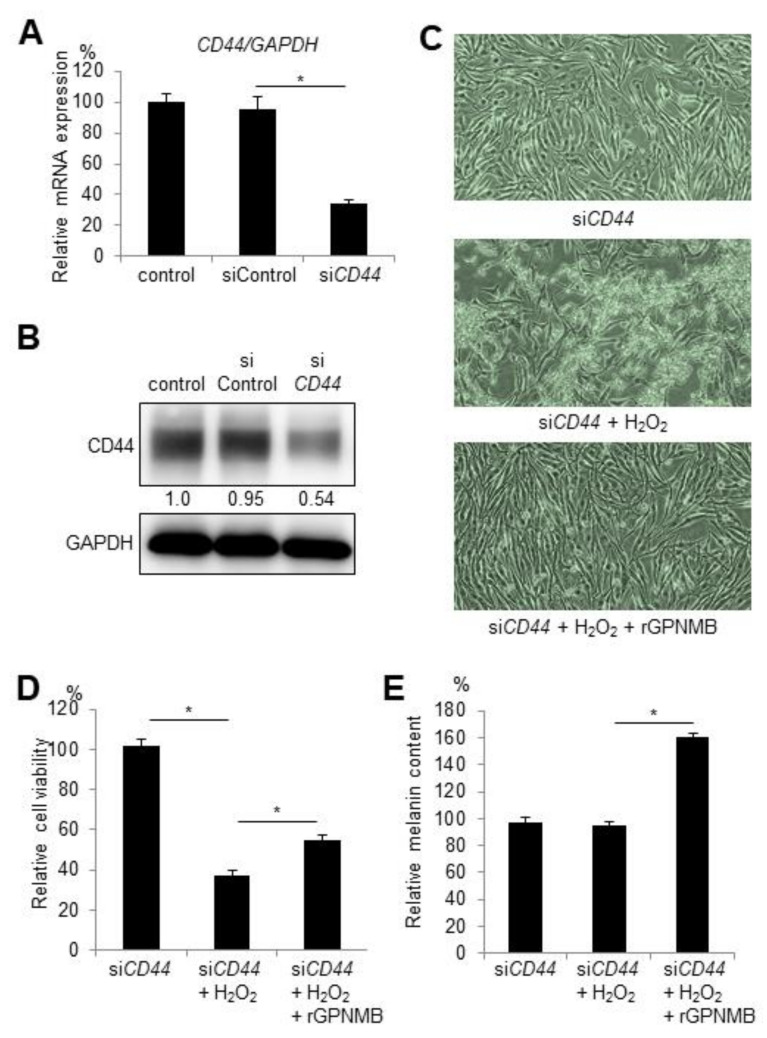
siRNA-mediated knockdown of CD44 did not alter the protective effect of sGPNMB in melanocytes. HEM-MP cells were transfected with control siRNA or *CD44* siRNA. (**A**) *CD44* mRNA expression was detected by qRT-PCR. (**B**) CD44 protein expression in cell lysates was analyzed by Western blotting. (**C**–**E**) After siRNA transfection, HEM-MP cells were treated with 0.2 mM of H_2_O_2_ and 500 ng/mL of rGPNMB for 48 h. (**C**) Cell shape was observed under a bright-field microscope. (**D**) Cell viability was quantified. (**E**) Melanin content in cell lysates was quantified. * *p* < 0.05 (one-way ANOVA with Tukey’s test).

Other studies have found that sGPNMB mediates signal transduction via cell surface proteins, such as CD44, which serve as receptors for GPNMB, showing neuroprotective effects [19]. To investigate whether CD44 is a possible receptor for sGPNMB binding in melanocytes, we knocked down CD44 in HEM-MP cells and examined the protective effect of rGPNMB. After *CD44* siRNA transfection into HEM-MP cells, both the mRNA and protein levels of CD44 were significantly downregulated (Figure 2A,B). HEM-MP cells were transfected with *CD44* siRNA and then treated with 0.2 mM of H_2_O_2_ and 500 ng/mL of rGPNMB. *CD44* silencing did not affect cell morphology or viability (data not shown). In addition, the protective effect of rGPNMB was not diminished with decreased CD44 expression (Figure 2C,D). Melanin biosynthesis was also activated after rGPNMB exposure following CD44 knockdown (Figure 2E). Therefore, the results showed that the protective effect of sGPNMB was independent of CD44 in melanocytes.

### 2.4. rGPNMB Dampened AKT Phosphorylation Induced by Oxidative Stress

**Figure 3:**
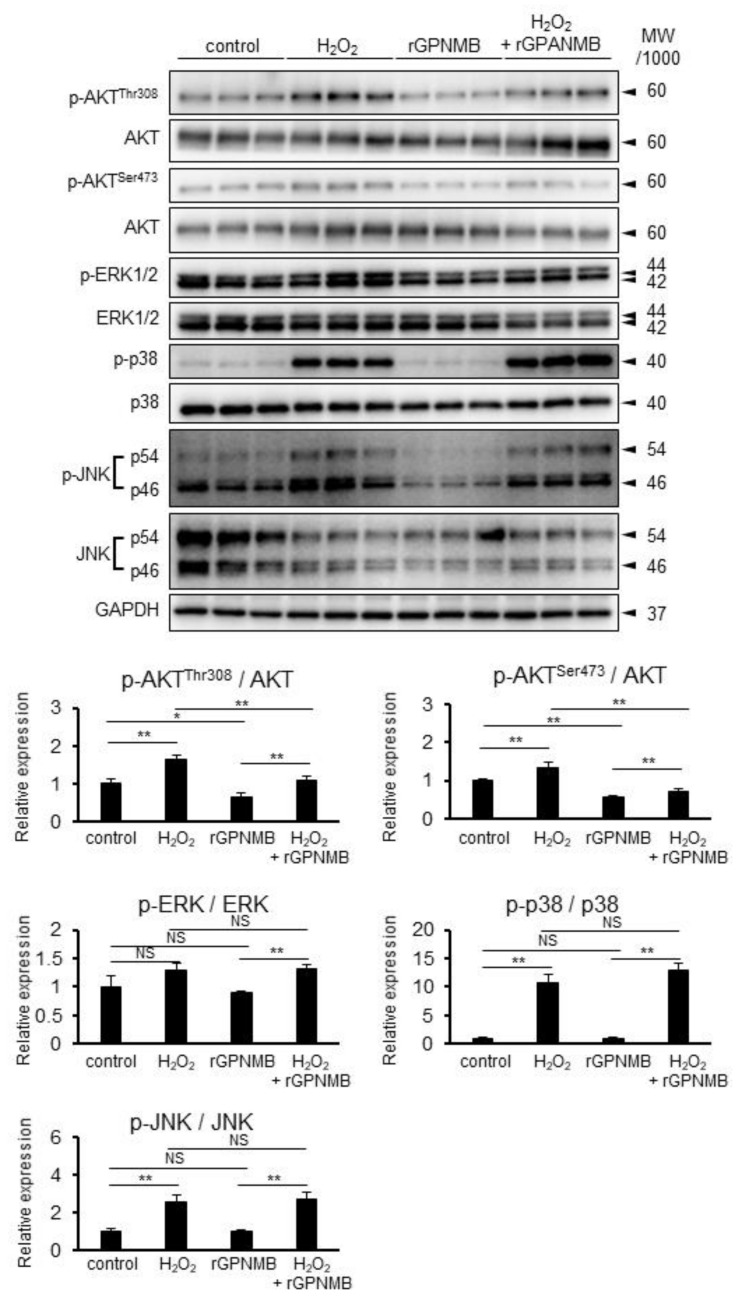
rGPNMB dampened AKT phosphorylation induced by oxidative stress in melanocytes. HEM-MP cells were treated with 0.4 mM of H_2_O_2_ and 500 ng/mL of rGPNMB separately or in combination for 60 min. Phosphorylated p-AKT-ser473, p-AKT-thr308, p-ERK, p-p38, p-JNK, total AKT, ERK, p38, JNK, and GAPDH protein expression in cell lysates were analyzed by Western blotting. The relative band intensity was calculated from the above Western blot data. * *p* < 0.05, ** *p* < 0.01 (one-way ANOVA with Tukey’s test). NS: not significant.

The phosphatidylinositol 3-kinase (PI3K)/AKT pathway and mitogen-activated protein kinase (MAPK)/extracellular signal-regulated kinase (ERK), p38, and c-Jun N-terminal kinase (JNK) pathways are the major factors related to cell survival and apoptosis [26,27]. H_2_O_2_ was found to increase p-AKT-Ser473, p-AKT-Thr308, p-ERK1/2-Thr202/Tyr204, p-p38 MAPK-Thr180/Tyr182, and p-JNK-Thr183/Tyr185 (Figure 3). However, rGPNMB decreased only pAKT-ser473 and pAKT-thr308, but not the other phosphorylated kinases. In addition, rGPNMB significantly suppressed the AKT phosphorylation induced by H_2_O_2_. Therefore, sGPNMB can selectively reduce the PI3K/AKT pathway.

### 2.5. GPNMB Expression Is Suppressed under Stress Conditions in Human Epidermal Keratinocytes

**Figure 4:**
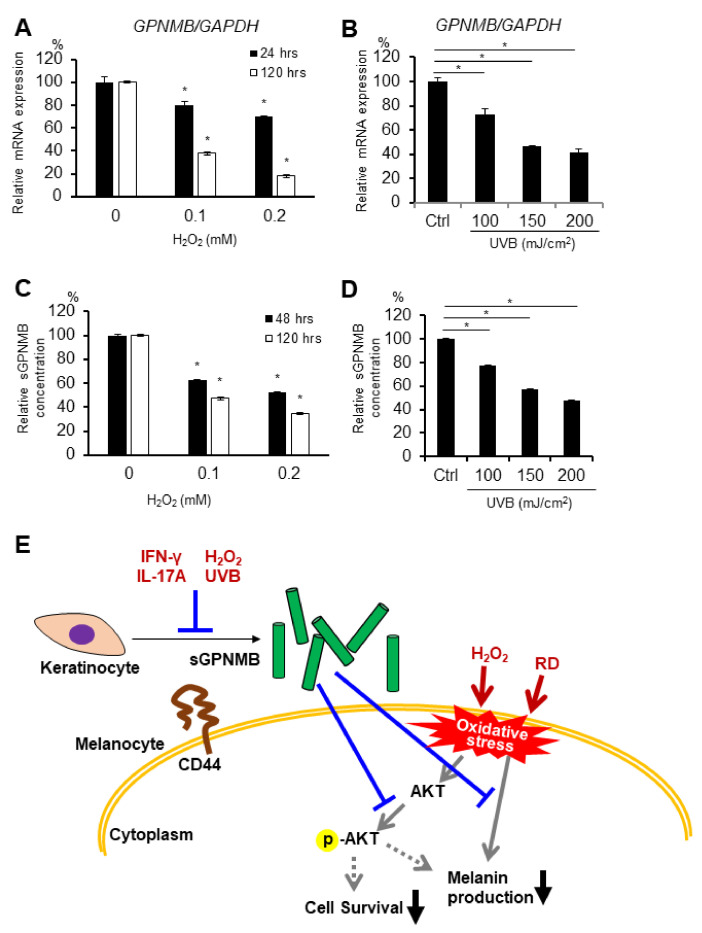
GPNMB expression was decreased by oxidative stress in PSVK1. PSVK1 was treated with the indicated concentration of H_2_O_2_ (**A**,**C**) or the indicated dose of UVB irradiation (**B**,**D**), then *GPNMB* mRNA expression was detected 24 h and 120 h after H_2_O_2_ exposure or UVB irradiation. (**A**,**B**) Released sGPNMB proteins were quantified after 48 h or 120 h after H_2_O_2_ exposure or UVB irradiation (**C**,**D**). * *p* < 0.05 vs. control (one-way ANOVA with Dunnett’s test) (**E**) Summary illustration of the possible role of sGPNMB and its involvement in vitiligo pathology. * *p* < 0.05 (one-way ANOVA with Dunnett’s test).

To examine whether GPNMB is expressed in skin cells, the *GPNMB* mRNA expression in 13 cell types (keratinocytes, melanocytes, fibroblasts, endothelial cells, skeletal muscle cells, and skin cancer cells) was analyzed using qRT-PCR. *GPNMB* was found to be expressed in primary melanocytes, melanoma cell lines, and keratinocytes that contained PSVK1—i.e., human primary epidermal keratinocytes from adults and newborns (Supplementary Figure S2). Hence, we used PSVK1 as the keratinocyte model. We previously showed that the possible causative cytokines in vitiligo development inhibited GPNMB expression in primary keratinocytes in vitro [9]. Interestingly, we also found that UVB irradiation and rhododendrol treatment (widely used oxidative stress inducers) slightly suppressed the phosphorylation of AKT (Figure S3), while H_2_O_2_ and UVB irradiation significantly decreased the *GPNMB* mRNA expression in PSVK1 cells (Figure 4A,B). Moreover, sGPNMB protein secretion decreased in the culture supernatants under both stress conditions in a dose-dependent manner (Figure 4C,D).

## 3. Discussion

GPNMB has been reported to have anti-inflammatory and neuroprotective functions in the brain [18,19] and to be associated with several neurodegenerative diseases (asemyotrophic lateral sclerosis and Alzheimer’s disease) [17,28]. In this study, we found that GPNMB could dampen cytotoxicity and inhibit melanogenesis against oxidative stress in melanocytes. GPNMB protects the melanocytes from oxidative stress under physiological conditions. The NRF2/HO-1 pathway has been reported as a classical pathway for oxidative stress [29]. However, the protective role of GPNMB has not been associated with NRF2/HO-1 expression. GPNMB is well known to be a melanosome-specific melanocyte marker that plays a critical role in melanosome formation [10,21]. Our data suggest that extracellular GPNMB increases melanin production. The silencing of *GPNMB* expression downregulated Pmel17 and TRP1 expression in the PIG1 melanocyte cell line [21]. Hence, both melanocyte-derived GPNMB and keratinocyte-derived GPNMB may contribute to melanosome formation. These effects can be induced by the extracellular domain of soluble GPNMB and membrane-bound GPNMB, both of which are expressed in keratinocytes.

In this study, we also identified diminished GPNMB expression in the epidermis of patients with RD-induced leukoderma. Furthermore, GPNMB was found to reduce RD cytotoxicity in melanocytes. When glutathione levels were decreased, RD metabolites (RD-quinone and RD-melanine) augmented melanocyte toxicity via oxidative stress [25,30]. That is, the reduction in GPNMB expression led to the attenuation of tolerance to RD metabolites.

To further explore the possible mechanism by which sGPNMB protects melanocytes from oxidative stress, we speculated that the receptor for the extracellular part of GPNMB could be expressed in melanocytes. Another study has reported that the extracellular part of GPNMB attenuated astrocyte-mediated neuroinflammation in a CD44-dependent manner [19], showing neuroprotective properties. However, after the siRNA-mediated knockdown of CD44 in melanocytes, the protective effect of sGPNMB was not altered. Interestingly, GPNMB accelerated melanin production, especially in the CD44 knockdown condition. It is possible that CD44 acts as a decoy receptor for extracellular GPNMB. Except for CD44, the possible receptors for GPNMB are αβ-integrins, the α1 subunit of Na^+^/K^+^-ATPase, FGFR1, and heparin sulfate proteoglycans [18,31,32,33]. Further studies are needed to define the receptors responsible for the protective role of GPNMB.

AKT is an important signaling kinase for cell survival in multiple systems. The phosphorylation of AKT was increased by H_2_O_2_ exposure, UVB irradiation, and rhododendrol treatment but suppressed by rGPNMB. AKT activation promotes mTOR signaling and inhibits autophagy [34]. Therefore, the inhibition of AKT phosphorylation by GPNMB can suppress proliferation and mTOR activation, which results in autophagy activation and the suppression of cell death. Nogueira et al. reported that the activation of AKT is susceptible to cell death induced by oxidative stress [35]. Therefore, GPNMB may inhibit cell damage from oxidative stress via the suppression of elevated AKT phosphorylation. The PI3K inhibitor LY294002 increases MITF expression and promotes melanogenesis [36]. In contrast, Haginin A, which had been isolated from the *Lespedeza cyrtobotrya* branch, inhibits melanogenesis via the downregulation of MITF and the activation of ERK and AKT/PKB [37]. LY294002 treatment recovered melanogenesis under the same conditions [37]. Therefore, GPNMB can augment melanogenesis via the increased expression of MITF and related melanogenic factors induced by AKT inactivation. In vitiligo patients, the phosphorylation level of AKT was lower in the lesional epidermis, whereas the expression level of PTEN was higher in the lesional epidermis than in the normally pigmented epidermis [38]. However, the levels of these proteins in vitiligo melanocytes remain unknown. The phosphorylation of MAPK (ERK, p38, and JNK) was increased by H_2_O_2_ exposure. Previous studies have shown that the phosphorylation of ERK, p38, and JNK is promoted in the presence of H_2_O_2_ in human melanocytes [39,40]. However, GPNMB did not affect the phosphorylation of these kinases. It has been demonstrated that the ERK pathway is significant for cell survival, whereas the p38 and JNK pathways are considered to be stress responsive and thus involved in apoptosis [27]. This means that GPNMB does not amplify cell survival signals and attenuates the stress-induced apoptosis pathway. Rather, GPNMB may play a major role in autophagy activation by suppressing AKT phosphorylation, which results in cell survival. In the present study, UVB irradiation and rhododendrol-induced AKT phosphorylation were only slightly suppressed by rGPNMB; the suppression effect on H_2_O_2_-induced AKT phosphorylation was much greater. This difference might have resulted from the complicated cellular responses in UVB irradiation and rhododendrol treatment besides oxidative stress.

We have previously shown that IFN-γ and IL-17A, which have also been reported as possible causative cytokines in vitiligo development, inhibited GPNMB expression [9]. In addition, oxidative stress (H_2_O_2_ and UVB) decreased both *GPNMB* mRNA expression and sGPNMB protein expression in cultured PSVK1 cells. Oxidative stress is one of the possible pathogenetic factors of vitiligo [41,42,43], and decreasing GPNMB expression in vitiligo is reasonable. Therefore, a combination of oxidative stress and these cytokines could suppress GPNMB expression in epidermal keratinocytes in patients with vitiligo. A small number of patients with RD-induced leukoderma could be coincident with vitiligo or have vitiligo triggered by RD-induced leukoderma [3]. It seems difficult to distinguish between vitiligo and RD-induced leukoderma on the basis of the clinical characteristics and histopathological findings. Further studies are needed to determine whether GPNMB expression is influenced by RD.

In summary, the study presented herein demonstrates that, in addition to melanocytes, epidermal keratinocytes express GPNMB, which can protect melanocytes from oxidative stress independent of the CD44 and NRF2/HO-1 pathways via AKT phosphorylation. Furthermore, IFN-γ, IL-17A, and oxidative stress dampened the GPNMB expression in vitiligo keratinocytes. The current study of GPNMB provides novel insights into the mechanisms associated with vitiligo pathology. It suggests that a decreased expression of GPNMB in the epidermis may be involved in the pathogenesis of vitiligo, and that approaches that reverse GPNMB expression in the epidermis may be effective in vitiligo treatments. Our results may aid in the development of new therapeutic strategies for vitiligo.

## 4. Materials and Methods

### 4.1. Human Skin Specimens

Skin samples were fixed in 10% formaldehyde for paraffin embedding. Paraffin-embedded tissue sections (3 μm) of lesional and perilesional skin from patients with RD-induced leukoderma were used in this study. Written informed consent was obtained from all the participants prior to inclusion in the study. The study was approved by the ethics committee of the Osaka City University Faculty of Medicine (No. 4152).

### 4.2. Immunohistochemistry

Paraffin sections were deparaffinized and heated at 95 °C for 16 min in Target Retrieval Solution (pH 9) for antigen retrieval. Tissue sections were then incubated with the primary and secondary antibodies listed below for 1 h at room temperature or overnight at 4 °C. The primary and secondary antibodies used are as follows: anti-human GPNMB (1:200; Sigma, St. Louis, MO, USA), anti-human Melan A (Agilent, Santa Clara, CA, USA), and Alexa 555–conjugated anti-rabbit or mouse secondary antibodies (1:500; Thermo Fisher Scientific, Waltham, MA, USA). Sections were then counterstained with Hoechst 33,342 (1:500; Thermo Fisher). The stained proteins were visualized using a fluorescence microscope (Biozero; Keyence, Osaka, Japan).

### 4.3. Cell Culture

The human epidermal keratinocyte cell line, PSVK1, was purchased from JCRB Cell Bank (Osaka, Japan) and cultured in KGM-Gold (Lonza, Basel, Switzerland) at 37 °C in 5% (*v/v*) CO_2_. Primary human neonatal epidermal melanocytes from a moderately pigmented donor (HEM-MP) were purchased from Thermo Fisher Scientific and cultured in Medium 254 with the addition of 1% (*v/v*) HMGS (Thermo Fisher Scientific) at 37 °C in 5% (*v/v*) CO_2_. The cells were used at passages 6–8 and seeded in 6-well plates (5 × 10^5^ cells/well), unless otherwise noted. Cell morphology was observed using inverted microscopy (Olympus, Tokyo, Japan).

### 4.4. Reagents

Hydrogen peroxide (H_2_O_2_) was purchased from Fujifilm Wako Pure Chemical (Osaka, Japan). Rhododendrol (RD) was kindly provided by Kanebo Cosmetics Inc. (Tokyo, Japan). Recombinant human GPNMB protein (extracellular fragment: Fc chimera) was purchased from R&D Systems (Cat# 2550-AC, Minneapolis, MN, USA). The bioactivity of this commercial rGPNMB has been measured and widely used for in vitro and in vivo experiments [18,19].

### 4.5. UV Irradiation

Cultured melanocytes were harvested in a 35 mm culture dish, the medium was removed, and phosphate buffered saline (PBS) was added. The lids were removed and the cells in the culture dish were exposed to UVB radiation from a xenon chloride excimer lamp (TheraBeam UV308 mini; Ushio, Tokyo, Japan). After exposure to UVB radiation, the cells were cultured in medium for the indicated periods.

### 4.6. RNA Isolation and Real-Time RT-PCR Analysis

Total RNA was isolated from pellets using a Maxwell RSC simplyRNA Tissue Kit (Promega, Madison, WI, USA) following the manufacturer’s instructions. Total RNA (200 ng) was reverse-transcribed into first-strand cDNA (ReverTra Ace qPCR RT Master Mix; TOYOBO, Osaka, Japan). The primers used for real-time PCR were as follows: *GPNMB*: 5’-GCGAGATCACCCAGAACACA-3’ (sense) and 5’-AGAGCCAGGCTTGTGTCATC-3’ (antisense); *GAPDH*: 5’-GACAGTCAGCCGCATCTTCT-3’ (sense) and 5’-GCGCCCAATACGACCAAATC-3’ (antisense); *PMEL17*: 5’-CTATGTGCCTCTTGCTCATTCC-3’ (sense) and 5’-TGCTTGTTCCCTCCATCCA-3’ (antisense); *TYR*: 5’-TGACTCCAATTAGCCAGTTCCT-3’ (sense) and 5’-GACAGCATTCCTTCTCCATCAG-3’ (antisense); *TYRP1*: 5’-CTCAATGGCGAGTGGTCTGT-3’ (sense) and 5’-TTCCAAGCACTGAGCGACAT-3’ (antisense); *CD44*: 5’-GATCACCGACAGCACAGACA-3’ (sense) and 5’-GCCATTCTGGAATTTGGGGTG-3’ (antisense). Each reaction was performed in triplicate.

### 4.7. ELISA Analysis

The concentration of GPNMB in the cell supernatants was determined using a commercial sandwich Human ELISA Kit (ab193711; Abcam, Cambridge, UK) according to the manufacturer’s instructions.

### 4.8. Cell Viability Assay

Melanocytes (1 × 10^4^ cells/well) were cultured in 96-well flat-bottom culture plates. After experimental treatments, cells were washed three times in cold PBS and cell viability was evaluated using the Cell Count Reagent SF colorimetric assay (Nacalai Tesque, Kyoto, Japan). Briefly, reagent (10 μL) was added to each well and the cells were incubated for 2 h at 37 °C. Cell viability was determined colorimetrically by measuring OD450 using a microplate reader (iMark; Bio-Rad Laboratories, Hercules, CA, USA). The ratio of absorbance in each treated group to the absorbance in the control group was calculated as the cell viability.

### 4.9. Melanin Quantification Assay

Melanocytes were dissolved in 200 μL of 1 N NaOH (30 min, 100 °C) and the concentration of melanin was quantified by measuring the absorbance at 405 nm, as described previously [44]. The calculated melanin content was adjusted based on the cell number.

### 4.10. Western Blot Analysis

Proteins from cells were extracted using lysis buffer 6 (R&D Systems), using 5 μg for Western blot analysis, as described previously [44]. The primary antibodies and dilutions used were as follows: anti-GPNMB (Sigma), 1:500; anti-GAPDH (Santa Cruz Biotechnology, Santa Cruz, CA, USA), 1:100; anti-CD44 (Cell Signaling Technology, Beverly, MA, USA), 1:1000; anti-pAKT-ser473 (Cell Signaling Technology), 1:2000; anti-pAKT-thr308 (Cell Signaling Technology), 1:1000; anti-AKT (Cell Signaling Technology), anti-pERK (Cell Signaling Technology), 1:1000, anti-ERK (Cell Signaling Technology), 1:1000, anti-p-p38 MAPK (Cell Signaling Technology), 1:1000; anti-p38 MAPK (Cell Signaling Technology), 1:1000; anti-p-SAPK/JNK (Cell Signaling Technology); 1:1000; and anti-SAPK/JNK (Cell Signaling Technology), 1:1000. GAPDH was used as a loading control. The signal intensity of bands was quantified using the Image J densitometry software (http://rsb.info.nih.gov/ij/index.html accessed on 31 August 2021) and normalized to the GAPDH signal intensity. Fold change was calculated as the ratio of each treatment to the control.

### 4.11. RNA Interference

For the small interfering RNA (siRNA)-mediated knockdown of CD44, melanocytes were transfected with either *CD44* or control siRNA (Thermo Fisher Scientific) using Lipofectamine^®^ RNAi MAX (Thermo Fisher Scientific) according to the manufacturer’s instructions. After the validation of the CD44 knockdown in the absence of effects on cell viability, functional assays were performed. The siRNA sequences targeting human *CD44* were designed using Thermo Fisher Scientific (NO. HSS101596).

### 4.12. Statistical Analysis

Data are expressed as mean ± SD. Two-group differences analyses were conducted using an unpaired Student’s *t*-test. The multi-group differences analyses were conducted using one-way ANOVA with Dunnett’s or Tukey’s comparison test. Statistical significance was set at *p* < 0.05. All statistical analyses and graphical representations of the data were performed and created using the EZR software (Version 1.40, Saitama Medical Center, Jichi Medical University, Saitama, Japan).

## Data Availability

The data presented in this study are available upon request from the corresponding author.

## Supplementary Materials

The following are available online at https://www.mdpi.com/article/10.3390/ijms221910843/s1: Figure S1: sGPNMB did not alter the endogenous antioxidant response in melanocytes under oxidative stress. Figure S2: mRNA expression levels of GPNMB in 13 types of skin cells were analyzed by quantitative real-time PCR. Figure S3: rGPNMB dampened AKT phosphorylation was induced by UVB and rhododendrol in melanocytes.
