# Supplementary figures and images for: GPNMB Extracellular Fragment Protects Melanocytes from Oxidative Stress by Inhibiting AKT Phosphorylation Independent of CD44
Source: Int J Mol Sci. 2021 Oct 7;22(19):10843. doi: 10.3390/ijms221910843 (PMC8509362; doi:10.3390/ijms221910843)

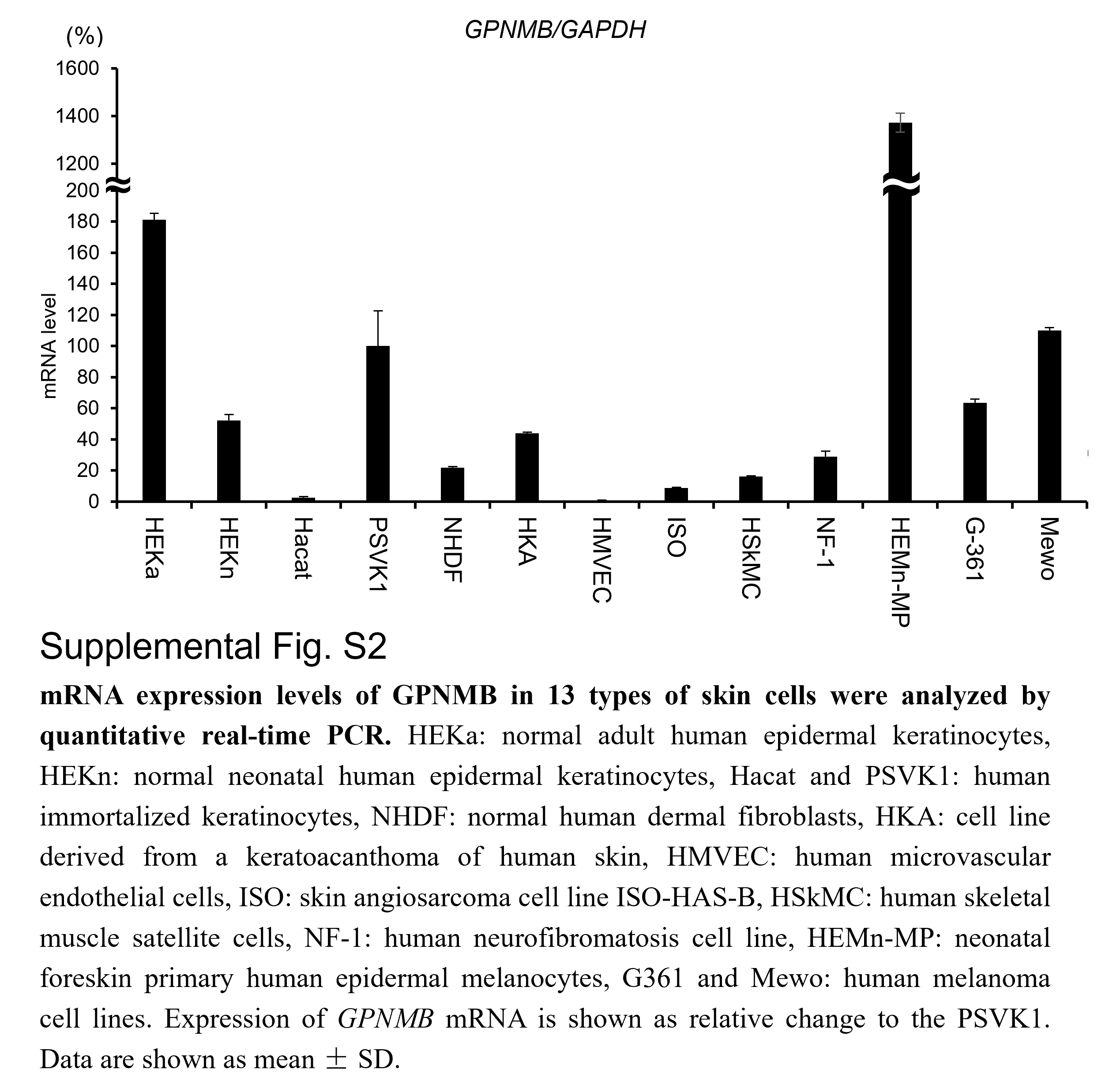

Supplement: Supplementary file 1 [file ijms-22-10843-s001.zip › Figure S2.tif]

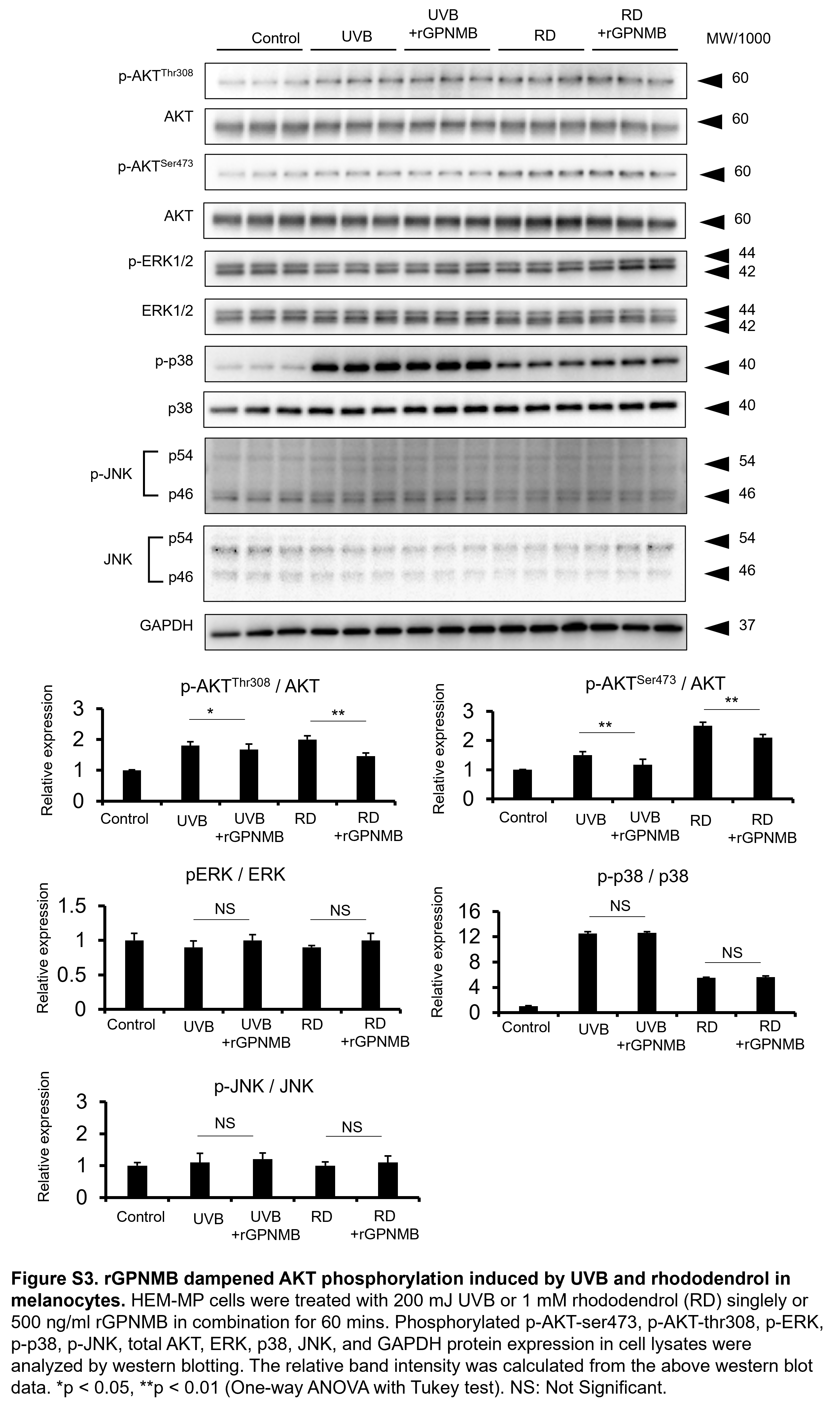

Supplement: Supplementary file 1 [file ijms-22-10843-s001.zip › Figure S3.tif]

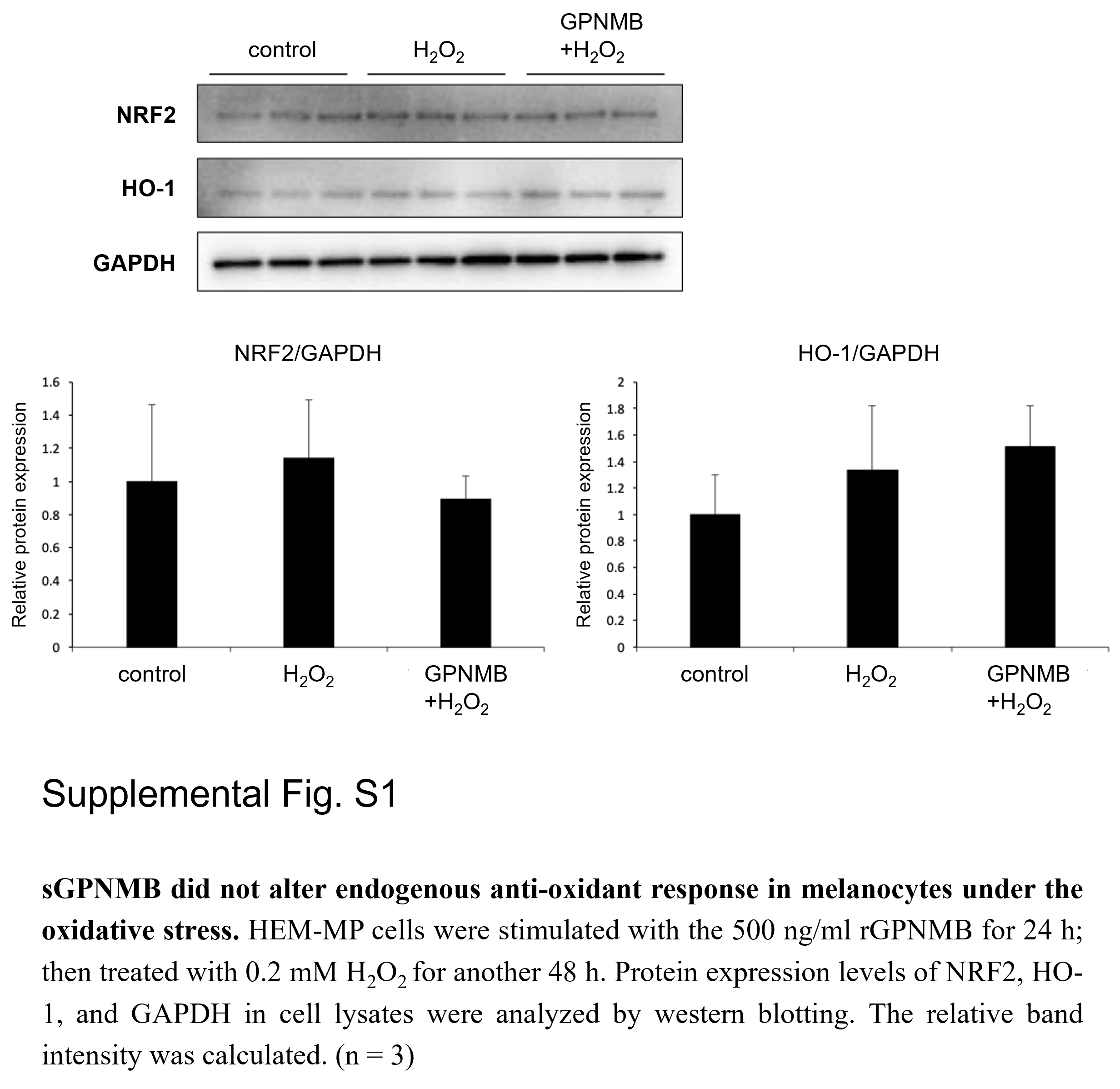

Supplement: Supplementary file 1 [file ijms-22-10843-s001.zip › Figure S1.tif]
